# Using the preoperative prognostic nutritional index as a predictive factor for non-cancer-related death in post-curative resection gastric cancer patients: a retrospective cohort study

**DOI:** 10.1186/s12876-020-01402-z

**Published:** 2020-08-05

**Authors:** Hitomi Takechi, Nobuaki Fujikuni, Kazuaki Tanabe, Minoru Hattori, Hironobu Amano, Toshio Noriyuki, Masahiro Nakahara

**Affiliations:** 1 Department of Surgery, Onomichi General Hospital, 1-10-23, Hirahara, Onomichi-shi, Hiroshima, 722-8508 Japan; 2 Department of Surgery, Division of Frontier Medical Science, Graduate School of Biomedical Sciences Hiroshima University, Hiroshima, Japan; 3 Center for Medical Education Institute of Biomedical & Health Sciences, Hiroshima University, Hiroshima, Japan

**Keywords:** Gastric cancer, Prognostic nutritional index, Cancer stage, Non-cancer-related death, Modified Glasgow prognostic score, Neutrophil-to-lymphocyte ratio, Kaplan-Meier curve, Gastrectomy, Overall survival, Recurrence-free survival

## Abstract

**Background:**

Gastric cancer (GC) is the third leading cause of cancer-related mortality worldwide. Therefore, identifying the predictive factors for surgical morbidity, disease recurrence, and long-term survival is necessary for preventing GC patient mortality. We aimed to evaluate the factors that contribute to the poor prognoses of GC patients.

**Methods:**

In this retrospective cohort study, the data of 182 patients who underwent curative gastrectomy for GC was reviewed. The data included patients’ cancer stage and preoperative prognostic nutritional index (PNI) score. We identified the prognostic factors using a univariate analysis and the multivariable Cox proportional hazards model. The associations between PNI and other clinicopathologic factors for GC were compared via logistic regression analysis. Kaplan-Meier curves were used to evaluate patients’ survival in relation to these factors. The median follow-up period was 3.5 years. Multivariable cumulative incidence method based on Fine and Gray’s method was performed to evaluate the association between non GC-related death and potential prognostic factors.

**Results:**

There were significant differences in overall survival (OS) between comorbidities (myocardial infarction: *P* = 0.040, liver disease: *P* = 0.017), cancer stages (I vs. II: *P* = 0.049, I vs. III: *P* < 0.001), tumor size (*P* = 0.002), lymphatic vessel infiltration (*P* < 0.001), serum CA 19–9 (*P* = 0.024), and PNI scores (*P* = 0.002). Moreover, only PNI score was determined to be an independent prognostic factor for survival. Furthermore, stage I GC patients with high PNI scores had significantly longer OS than those with low PNI scores (*P* < 0.001), but these groups were not significantly different in terms of recurrence-free survival (*P* = 0.756). Stage II and III GC patients showed no significant difference in terms of OS and recurrence-free survival, regardless of PNI scores. Finally, Fine and Gray’s method revealed that PNI score was an independent prognostic factor for non-GC-related death (*P* < 0.001).

**Conclusions:**

Preoperative PNI is effective in predicting the prognosis of post-curative gastrectomy GC patients and can be used to predict non-GC-related death and the OS of post-curative gastrectomy patients with stage I GC.

## Background

Gastric cancer (GC) is one of the most common malignancies and the third leading cause of cancer-related deaths worldwide. There were 782,685 GC deaths, constituting approximately 8.2% of the total cancer deaths among 185 countries in 2018 [1]. Thus, there has been increased interest in the prognostic factors that can accurately identify patients with a high risk of cancer recurrence and death. Identifying the predictive factors for surgical morbidity, disease recurrence, and long-term survival in GC patients can enhance the effectiveness of individualized perioperative management in preventing patient mortality. The relationship between malnutrition evaluated via the prognostic nutritional index (PNI) and patient outcomes has been well-established in GC cases [2, 3]. Moreover, the relationship between patient outcomes and various other prognostic factors, such as the modified Glasgow Prognostic Score (mGPS), a score that is based on serum biomarkers and used to evaluate the prognoses of cancer patients, and neutrophil-to-lymphocyte ratio (NLR), has also been investigated [4–7]. We aimed to evaluate the factors that contribute to the poor prognoses of GC patients in our hospital.

## Methods

### Study design

**Fig. 1:**
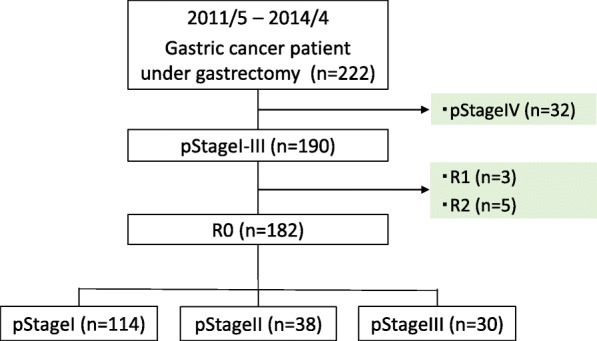
Study profile

We conducted a retrospective cohort study where we reviewed the medical records of 222 GC patients who underwent gastrectomy between May 2011 and April 2014 at Onomichi General Hospital, Hiroshima, Japan. Thirty-two patients with Stage IV were ineligible. All patients were histologically confirmed to have stage I, II, or III gastric adenocarcinomas via the Japanese classification of gastric carcinoma: 3rd English edition [8]. From these patients we selected who had R0 operation; 3 patients with microscopically incomplete resection (R1) or 5 patients with macroscopically incomplete resection (R2) were excluded. The study profile is shown in Fig. 1.

Almost all patients underwent total or distal gastrectomy and D1+ or D2 lymph node dissection in accordance with the Japanese Gastric Cancer Treatment Guidelines published in 2010 (ver. 3) [9]. The patients’ medical records were reviewed to gather data, including age, gender, body mass index, comorbidities, preoperative laboratory test results (absolute neutrophil and lymphocyte counts, and serum albumin and C-reactive protein (CRP) concentrations), surgical procedures, surgical pathology reports, and survival times.

In accordance with current clinical guidelines, postoperative patients with stage II and III GC and no marked comorbidities that would preclude chemotherapy use, were offered 5-fluorouracil-based adjuvant chemotherapy [10] after discussing with the patients and their family members. Postoperative follow-ups, which included physical examinations, laboratory tests, chest and abdominal cavity enhanced computed tomography, and upper gastrointestinal endoscopy, were conducted within 5 years post-operation, or until death, in accordance with the Japanese Gastric Cancer Treatment Guidelines [9]. The latest follow-up was on March 2017, and the median follow-up period was 39 months (range, 1–72). Overall survival (OS) was defined as the time between the date of surgery and death or the last available follow-up, and recurrence-free survival (RFS) was defined as the time between the date of surgery and disease recurrence or the last available follow-up.

### Complications

The Clavien-Dindo classification system was applied in grading the postoperative complications [11]. These grades are as follows: grade 1 (any deviation from the normal postoperative course without the need for pharmacologic treatment or surgical, endoscopic, or radiologic interventions), grade 2 (pharmacologic treatment required), grade 3 (surgical, endoscopic, or radiologic intervention required), grade 4 (life-threatening complications requiring intensive care unit (ICU) management), and grade 5 (patient death). Complications classified as grade 3 or higher were considered major.

### Investigational variables

Routine blood and biochemical tests were conducted on the day before surgery to obtain patients’ absolute neutrophil and lymphocyte counts, and serum albumin and CRP concentrations. Patients’ mGPS was evaluated, and patients with high CRP levels (> 0.5 mg/dL) and hypoalbuminemia (< 3.5 g/dL) were given an mGPS of 2. Patients exhibiting only one of these parameters were given an mGPS of 1. Patients exhibiting neither of these parameters were given an mGPS of 0 [12]. After NLRs were calculated, we defined a low NLR as an NLR <  2.5 and a high NLR as an NLR ≥ 2.5 [13]. Patients were divided into either the low or high NLR group. The PNI score was calculated using the following formula: 10 × serum albumin concentration (g/dL) + 0.005 × lymphocyte count (number/mm^2^), as proposed by Onodera et al. [14] Generally, the resection and anastomosis of gastrointestinal tracts can be performed safely on patients with a PNI score of over 45, but these procedures may be dangerous for patients with a PNI score between 40 and 45. Additionally, these procedures may be contraindicated for patients with a PNI score of less than 40. Therefore, we defined a low PNI score as a score <  45 and a high PNI score as a score ≥ 45 [15]. Patients were also divided into either the low or high PNI group.

Tumor markers, including serum carcinoembryonic antigen (CEA) and carbohydrate antigen 19–9 (CA19–9), were examined preoperatively. The cut-off points for normal serum levels were 5.5 U/dL for CEA and 37 U/dL for CA19–9.

### Statistical analyses

**Fig. 2:**
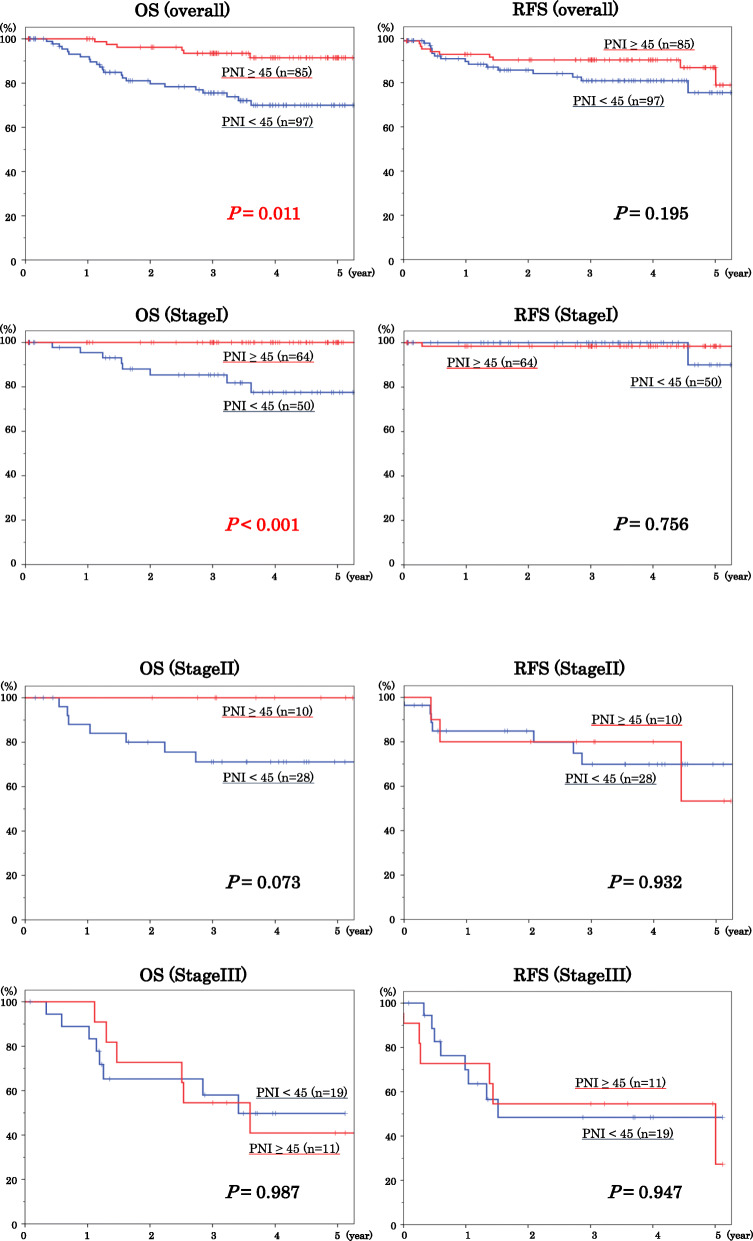
Survival curves in PNI groups by stage indicate the overall survival (OS) in the left line, and the recurrence-free survival (RFS) in the right line

A simple univariate logistic regression analysis was used to evaluate the association between PNI score and other clinicopathologic factors. The Kaplan-Meier method was used to estimate cumulative survival and assess the relationship between survival and prognostic factors (Fig. 2), and the statistical significance of differences was assessed via the log-rank test. The multivariable Cox proportional hazard model was applied for variables that proved to be significant in a univariate analysis. These statistical analyses were performed using SPSS Statistics software (version 19; SPSS, Chicago, IL, USA). A *P*-value of < 0.05 was considered statistically significant. In addition, to assess the statistical significance of PNI as a prognostic factor for non-GC-related death, a cumulative incidence analysis was performed using Gray’s test. GC-related deaths were considered to be competing risk events because GC-related deaths prevented the occurrence of non-GC-related deaths. By calculating the cumulative incidence function, death was taken in to account as a competing risk factor. Cumulative incidence function was plotted for, both, the whole cohort and the subgroups stratified by pStage. The Fine and Gray model was used to measure the burden of cumulative incidence of non-GC-related death and strength of its association with potential prognostic factors. All variables were used to select the model using a stepwise method with Bayesian information criterion. These statistical analyses were performed using R software (version 3.25).

## Results

Patients’ demographic and oncological characteristics are shown in Table 1. There were 130 men (71.4%) and 52 women (28.6%) with a median age of 70 years (range, 38–90 years). Of these, 114 patients (62.6%) had stage I GC, 38 patients (20.9%) had stage II GC, and 30 patients (16.5%) had stage III GC. During the follow-up period, 29 patients (15.9%) died. All tumors were pathologically diagnosed as adenocarcinomas, with 102 patients (56.0%) having intestinal adenocarcinomas and 80 patients (44.0%) having diffuse adenocarcinomas. Distal gastrectomy was performed on 124 patients (68.1%); total gastrectomy on 51 patients (28.0%); and proximal gastrectomy on 7 patients (3.8%).

**Table 1:** General characteristics of 182 GC patients

Variables	Values
Age (years)	70 (38–90)
≥ 70 / < 70	92 (50.5%) / 90 (49.5%)
Sex	
male / female	130 (71.4%) / 52 (28.6%)
BMI (kg/m^2^)	22.7 (14.2–35.8)
≥ 25 / < 25	49 (26.9%) / 133 (73.1%)
Comorbidities	
Myocardial infarction	7 (3.8%)
Congestive heart failure	11 (6.0%)
Cerebrovascular accident	12 (6.6%)
COPD	14 (7.7%)
Liver disease	6 (3.3%)
Chronic kidney disease	2 (1.1%)
Diabetes mellitus	35 (19.2%)
Stage	
I / II / III	114 (62.6%) / 38 (20.9%) / 30 (16.5%)
Depth of tumor invasion (T)	
1 / 2 / 3 / 4	99 (54.4%) / 23 (12.6%) / 55 (30.2%) / 5 (2.7%)
Tumor size (mm)	40 (4–140)
≥ 50 / < 50	59 (32.4%) / 123 (67.6%)
Lymph node metastasis (N)	
0 / 1 / 2 / 3	127 (69.8%) / 25 (13.7%) / 13 (7.1%) / 17 (9.3%)
Lymphatic vessel infiltration	80 (44.0%)
Histologic type	
Intestinal / Diffuse	102 (56.0%) / 80 (44.0%)
Approach	
Open / Laparoscopic	93 (51.1%) / 89 (48.9%)
Operative method	
Distal / Total / Proximal	124 (68.1%) / 51 (28.0%) / 7 (3.8%)
Lymph node dissection	
D1 / D1+ / D2	32 (17.6%) / 74 (40.7%) / 76 (41.8%)
Complication (CD ≥ 3)	9 (4.9%)
Complication (infection)	14 (7.7%)
Length of stay (day)	13 (8–178)
≥ 20 / < 20	26 (14.3%) / 156 (85.7%)
Tumor markers	
CEA (ng/mL)	2.6 (0.5–68.8)
≥ 5.5 / < 5.5	18 (9.9%) / 164 (90.1%)
CA19–9 (ng/mL)	7.35 (0.1–1048.3)
≥ 37 / < 37	19 (10.4%) / 163 (89.6%)
mGPS	
0 / 1 / 2	167 (91.8%) / 7 (3.8%) / 8 (4.4%)
NLR	2.0 (0.56–15.37)
≥ 2.5 / < 2.5	59 (32.4%) / 123 (67.6%)
PNI	44.1 (26.0–64.7)
≥ 45 / < 45	85 (46.7%) / 97 (53.3%)
Adjuvant chemotherapy	33 (18.1%)

Data are median (range) or number (%). Some percentages do not add up to 100 because of rounding

*BMI* body mass index, *COPD* chronic obstructive pulmonary disease, *CD* Clavien-Dindo Classification, *CEA* carcinoembryonic antigen, *CA19–9* carbohydrate antigen 19–9, *mGPS* modified Glasgow Prognostic Score, *NLR* neutrophil-to-lymphocyte ratio, *PNI* prognostic nutritional index

The results of the univariate analysis of prognostic factors for OS are listed in Table 2. It shows that there were significant differences in OS between the comorbidities (myocardial infarction: *P* = 0.040, liver disease: *P* = 0.017), cancer stages (I vs. II: *P* = 0.049, I vs. III: *P* < 0.001), tumor size (*P* = 0.002), lymphatic vessel infiltration (*P* < 0.001), serum CA19–9 (*P* = 0.024), and PNI scores (*P* = 0.002). However, there were no significant differences in OS between different mGPSs (*P* = 0.364) and different NLRs (*P* = 0.669). Additionally, there was no significant difference in OS between serum CEA (*P* = 0.068). Furthermore, our multivariable analysis revealed that only PNI score was independent prognostic factor for OS (*P* = 0.031).

**Table 2:** Univariate and multivariate analysis of overall survival

Variables	Univariate analysis		Multivariate analysis	
	HR (95% CI)	*P* value	HR (95% CI)	*P* value
Age (≥ 70 vs < 70)	2.023 (0.955–4.287)	0.066		
Sex (female vs male)	1.195 (0.544–2.624)	0.658		
BMI (< 25 vs ≥ 25)	1.977 (0.754–5.183)	0.166		
Comorbidities				
Myocardial infarction	**3.510 (1.057–11.655)**	**0.040**	1.825 (0.520–6.413)	0.348
Congestive heart failure	2.848 (0.990–8.194)	0.052		
Cerebrovascular accident	1.330 (0.316–5.603)	0.697		
COPD	1.393 (0.331–5.858)	0.651		
Liver disease	**4.267 (1.291–14.108)**	**0.017**	3.514 (0.868–14.235)	0.078
Chronic kidney disease	6.615 (0.863–50.679)	0.069		
Diabetes mellitus	1.460 (0.647–3.297)	0.363		
Stage				
(II vs I)	**2.766 (1.003–7.629)**	**0.049**	1.211 (0.314–4.671)	0.781
(III vs I)	**8.214 (3.437–19.631)**	**< 0.001**	3.042 (0.784–11.812)	0.108
Tumor size (≥ 50 vs < 50)	**3.210 (1.543–6.678)**	**0.002**	1.080 (0.441–2.648)	0.866
Lymphatic vessel infiltration	**5.739 (2.335–14.104)**	**< 0.001**	3.054 (0.879–10.612)	0.079
Histologic type (Intestinal vs Diffuse)	1.089 (0.52–2.280)	0.821		
Lymph node dissection				
(D1+ vs D1)	3.760 (0.854–16.550)	0.080		
(D2 vs D1)	3.262 (0.736–14.459)	0.120		
Complication (CD ≥ 3)	1.659 (0.394–6.982)	0.490		
Complication (infection)	2.343 (0.815–6.739)	0.114		
CEA (≥ 5.5 vs < 5.5)	2.306 (0.939–5.665)	0.068		
**CA19–9 (**≥ **37 vs < 37)**	**2.825 (1.150–6.938)**	**0.024**	1.340 (0.453–3.961)	0.597
mGPS (0 vs 1,2)	2.519 (0.343–18.517)	0.364		
NLR (≥ 2.5 vs < 2.5)	1.182 (0.55–2.542)	0.669		
**PNI (< 45 vs ≥ 45)**	**4.261 (1.734–10.47)**	**0.002**	**2.889 (1.104–7.563)**	**0.031**

*HR* hazard ratio, *CI* confidential index. Variables in bold are statistically significant (*P* < 0.05)

*BMI* body mass index, *COPD* chronic obstructive pulmonary disease, *CD* Clavien-Dindo Classification, *CEA* carcinoembryonic antigen, *CA19–9* carbohydrate antigen 19–9, *mGPS* modified Glasgow Prognostic Score, *NLR* neutrophil-to-lymphocyte ratio, *PNI* prognostic nutritional index

Therefore, we investigated the association between PNI score and other clinicopathologic factors (Table 3) and our logistic regression analysis revealed that age (*P* < 0.001), comorbidities (congestive heart failure: *P* = 0.032 and chronic obstructive pulmonary disease (COPD): *P* = 0.023), cancer stage (*P* = 0.008), tumor size (*P* = 0.001), lymphatic vessel infiltration (*P* = 0.005), and lymph node dissection (*P* = 0.001) significantly correlated with PNI score. Furthermore, our Kaplan-Meier curves showed that, for all GC stages, the low PNI group was significantly associated with poor OS (*P* = 0.011), but PNI scores did not significantly correlate with RFS (*P* = 0.195). Furthermore, the survival curve of stage I GC patients in the high PNI group showed significantly better OS than that of patients in the low PNI group (*P* < 0.001), but there was no significant difference in RFS between those in either of the PNI groups (*P* = 0.756). For stage II GC patients, there was no significant difference in OS and RFS between the PNI groups (*P* = 0.073 and *P* = 0.932, respectively). However, none of the stage II GC patients in the high PNI group died within the study period. Finally, there was no significant difference in OS and RFS between the stage III GC patients of either PNI group (*P* = 0.987 and *P* = 0.947, respectively).

**Table 3:** Relationship between PNI and the clinicopathologic features

Variables		PNI < 45 (*n* = 97)	PNI ≥ 45 (*n* = 85)	*p* value
Age	< 70	13 (13.4%)	77 (90.6%)	**< 0.001**
	≥ 70	84 (86.6%)	8 (9.4%)	
Sex	Male	70 (72.2%)	60 (70.6%)	0.814
	Female	27 (27.8%)	25 (29.4%)	
BMI	< 25	74 (76.3%)	59 (69.4%)	0.298
	≥ 25	23 (23.7%)	26 (30.6%)	
Comorbidities				
Myocardial infarction	present	7 (7.2%)	0 (0.0%)	0.999
	absent	90 (92.8%)	85 (100.0%)	
Congestive heart failure	present	10 (10.3%)	1 (1.2%)	**0.032**
	absent	87 (89.7%)	84 (98.8%)	
Cerebrovascular accident	present	9 (9.3%)	3 (3.5%)	0.133
	absent	88 (90.7%)	82 (96.5%)	
COPD	present	12 (12.4%)	2 (2.4%)	**0.023**
	absent	85 (87.6%)	83 (97.6%)	
Liver disease	present	6 (6.2%)	0 (0.0%)	0.999
	absent	91 (93.8%)	85 (100.0%)	
Chronic kidney disease	present	2 (2.1%)	0 (0.0%)	0.999
	absent	95 (97.9%)	85 (100.0%)	
Diabetes mellitus	present	18 (18.6%)	17 (20.0%)	0.805
	absent	79 (81.4%)	68 (80.0%)	
Stage	I	50 (51.5%)	64 (75.3%)	**0.008**
	II	28 (28.9%)	10 (11.8%)	
	III	19 (19.6%)	11 (12.9%)	
Tumor size	50 >	55 (56.7%)	68 (80.0%)	**0.001**
	≥ 50	42 (43.3%)	17 (20.0%)	
Lymphatic vessel infiltration	present	52 (53.6%)	28 (32.9%)	**0.005**
	absent	45 (46.4%)	57 (67.1%)	
Histologic type	Intestinal	59 (60.8%)	43 (50.6%)	0.166
	Diffuse	38 (39.2%)	42 (49.4%)	
Operative method	Distal	68 (70.1%)	56 (65.9%)	0.330
	Total	27 (27.8%)	24 (28.2%)	
	Proximal	2 (2.1%)	5 (5.9%)	
Lymph node dissection	D1	4 (4.1%)	28 (32.9%)	**0.001**
	D1+	48 (49.5%)	26 (30.6%)	
	D2	45 (46.4%)	31 (36.5%)	
Complication (CD ≥ 3)	present	6 (6.2%)	3 (3.5%)	0.415
	absent	91 (93.8%)	82 (96.5%)	
Complication (infection)	present	11 (11.3%)	3 (3.5%)	0.062
	absent	86 (88.7%)	82 (96.5%)	

Data are presented as number (%). Variables in bold are statistically significant (*P* < 0.05)

*BMI* body mass index, *COPD* chronic obstructive pulmonary disease, *CD* Clavien-Dindo Classification

**Fig. 3:**
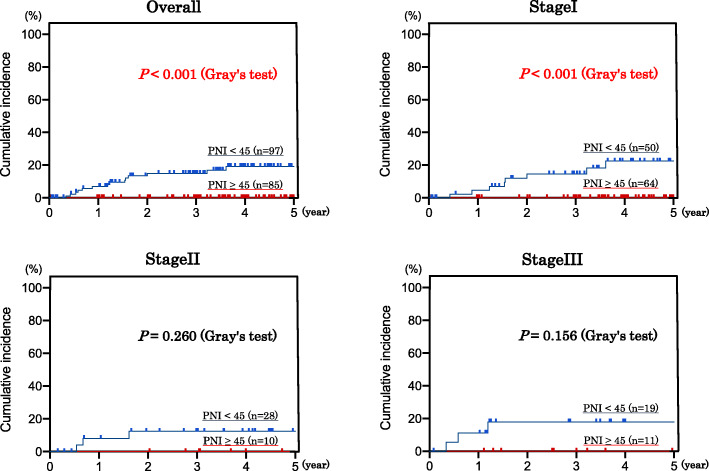
Cumulative Incidence of non-GC-related death by PNI

Finally, Gray’s test showed that when death was considered as a competing risk factor, the cumulative morbidity function of non-GC-related deaths was significantly different in the high PNI group than that in the low PNI group for all GC stages (*P* < 0.001) as well as stage I (*P* < 0.001) (Fig. 3). A multivariable cumulative incidence method based on the Fine and Gray test with non-GC-related death as a competing risk revealed that only PNI score was an independent prognostic factor for non-GC-related death (sHR = 1.29^e-05^ [95%CI 7.32^e06^ - 2.27^e-05^], *P* < 0.001). Conversely, the cumulative incidence method based on the Fine and Gray test, which focused on GC-related death, showed no significant difference in PNI, indicating that stage is an independent prognostic factor for GC-related death (sHR = 7.653 [95%CI 3.751–15.61], *P* < 0.001).

## Discussion

GC is one of the most common malignancies worldwide, but the results of GC treatment remain unpredictable. We have observed both long-term and short-term survivors, in spite of providing the same treatment. Therefore, to analyze the reasons for the differences in prognoses between patients, we evaluated clinicopathological factors that may significantly affect patient prognosis. The introduction of laboratory findings like preoperative serum tumor markers could provide prognostic information in patients with GC [16]. The mGPS and NLR are the inflammation-based biomarkers, and cancer-related inflammation has been shown to have adverse effects on cancer prognosis. The PNI is a nutritional status parameter. Multiple factors including cancer-associated malnutrition have been related to poor response to therapy. Thus, the identification of further sensitive prognostic markers in this subgroup may help in the better treatment for GC patients.

Our univariate analysis showed significant differences in terms of survival between comorbidities (myocardial infarction and liver disease), cancer stages, tumor size, lymphatic vessel infiltration, serum CA19–9, and PNI scores. After excluding correlated variables, the multivariable analysis showed that only PNI score was independent prognostic factor for survival. Therefore, we focused on the PNI and examined the association between PNI and other factors. Since PNI significantly correlated with age, congestive heart failure, COPD, cancer stage, tumor size, lymphatic vessel infiltration, and lymph node dissection, we examined the significance of PNI score for each cancer stage.

Initially, our Kaplan-Meier curves for all cancer stages showed that the low PNI group was significantly associated with poor OS, but there was no significant difference in RFS between either PNI groups. When we investigated each cancer stage in detail, we found there was no mortality in stage I GC patients with high PNI, however nearly 30% of those in the low PNI group died within the follow-up period. However, few stage I patients experienced cancer recurrence. Therefore, we believe that the PNI was a factor for non-GC-related deaths rather than a predictive factor for cancer recurrence in the stage I GC patients. The causes of death for these patients were heart failure, pneumonias, different type of cancer than GC, cirrhosis, and cerebral infarction. There was no GC-related death (Table 4). There were a few GC-related deaths in stage II and III GC patients, but non-GC-related deaths were observed only in the low PNI group. These observations indicated that the low PNI could be used as a prognostic marker to predict non-GC-related deaths regardless of GC stage. In addition, a multivariable cumulative incidence method based on Fine and Gray’s method also revealed the prognostic significance of PNI score in predicting non-GC-related death (*P* < 0.001).

**Table 4:** Cause of death

	PNI	Stage I		Stage II		Stage III	
		high	low	high	low	high	low
Total		0/64	8/50	0/10	7/28	6/11	8/19
GC-related death					4	6	5
Different type of cancer			1				1
Heart failure			1				
Pneumonia			4		2		1
Cirrhosis			1		1		
Cerebral infarction			1				
Cerebral hemorrhage							1

*PNI* prognostic nutritional index, *GC* gastric cancer

Moreover, all stage II GC patients in the high PNI group remained alive and their Kaplan-Meier curves were similar to those for stage I GC patients in terms of OS, but the recurrence rates between both PNI groups were similar. We believe that long-term survival was seen because the high PNI group could be safely treated with chemotherapy if their cancer recurred. However, stage II GC patients in the low PNI group exhibited poor OS because they could not undergo adjuvant chemotherapy or chemotherapy after recurrence. Due to their low vitality, active treatment interventions became difficult to implement and their OS was affected by cancer recurrence and non-GC-related death. Specifically, 90% of the high PNI group could be given chemotherapy, whereas only 46% of the low PNI group could be given chemotherapy.

Finally, there was no significant difference between the stage III GC patients of either PNI group in terms of OS or RFS. The main cause of death for stage III GC patients was GC and it was difficult to improve their OS by providing medical treatment, such as chemotherapy, when GC recurred.

In this study, we were able to use the PNI as a prognostic factor for GC patients who underwent R0 gastrectomy at our hospital. After the analysis of each individual cancer stage, our results showed that we could use the preoperative PNI to predict postoperative non-GC-related death, consider whether additional treatment, such as chemotherapy, can be administered, and evaluate patients’ potential for long-term postoperative survival. Moreover, a simple literature search using the terms “gastric cancer” and “PNI” on PubMed yielded 104 results as of February 2019 and none of them focused on postoperative non-GC-related death.

Because the PNI is an index of nutritional status, the PNI in cases of early GC and that in cases of advanced GC have different meanings. We speculate that a general status with comorbidities is reflected in the PNI of early GC patients because of less influence of tumor, but the aggravation of the general status is reflected in the PNI of advanced GC patients because of neoplastic hypoalimentation. Additionally, patients with GC are often malnourished [17], and poor oral nutritional intake and protein loss caused by primary lesions cause cancer cell to secrete cytokines, such as tumor necrosis factor-alpha, that adversely affect catabolic metabolism [18, 19]. That is, cancer stage has a correlative relation with the PNI score, and the PNI score is aggravated by cancer progression. Implementing intervention methods to improve patients’ OS using preoperative nourishment status as a guide is difficult because the time between identifying cancer and treatment operations is limited. Therefore, the PNI score will play a role only as a convalescence prediction marker. Even though a neo-adjuvant chemotherapy can improve the advanced GC stage, the performance status of the patients may worsen such that they cannot receive chemotherapy. Although this may be influenced by a deterioration in postoperative physical strength, there were many patients of the low PNI group who could not undergo the adjuvant chemotherapy in this study. Further study will be necessary to improve the perioperative PNI score.

Finally, the influence of inflammatory indexes such as mGPS and NLR on prognoses was unclear in this study. In addition to the nourishment status, neoplastic inflammation might also correlate with clinicopathological features in GC patients; there is a possibility that its influence was not significant because majority of the patients in the study cohort were stage I patients.

However, there are some limitations in the present study. One of the limitations is that calibration and concordance statistics have not been calculated. Moreover, the sHR for the PNI effect from the Fine and Gray Proportional Hazards regression has become quite huge. This may due to the fact that our study is a retrospective, single-center study with a small sample size and short follow-up time of only 3.5 years. Therefore further studies are required with large sample size and longer follow-up periods are required to confirm our results.

## Conclusions

The preoperative PNI was effective in predicting the prognosis of post-curative gastrectomy in GC patients in our study. Our study also suggests that we could use the preoperative PNI to predict postoperative non-GC-related death, and determine which patients have poor vitalities, in order to consider when to provide or withhold treatment. Moreover, it can predict the OS of post-curative gastrectomy patients with stage I GC. Future research regarding new methods that improve GC patients’ nutritional status is necessary to mitigate the limitations of using the preoperative PNI as a prognostic factor in GC patients.

## Data Availability

All data generated or analyzed during this study are included in this published article. The datasets used and/or analyzed during the current study are available from the corresponding author on reasonable request.

## Abbreviations

RFS: Recurrence-free survival
GC: Gastric cancer
mGPS: Modified Glasgow Prognostic Score
NLR: Neutrophil-to-leukocyte ratio
OS: Overall survival
PNI: Prognostic nutritional index
